# Serendipitous Manifestation of Intra-abdominal Mass Leading to Primary Gout Diagnosis: A Case Report

**DOI:** 10.7759/cureus.89735

**Published:** 2025-08-10

**Authors:** Siddhanth S Iyer, Su-hun Seo, Srinivas Iyer

**Affiliations:** 1 Radiology, George Washington University School of Medicine and Health Sciences, Washington D.C., USA; 2 Department of Radiology, University of Pittsburgh Medical Center, Pittsburgh, USA

**Keywords:** abdominal mass, celiac, extra-articular, gout, lymphoma

## Abstract

Gout is characterized by the deposition of monosodium urate (MSU) crystals with negative birefringence under polarized light in synovial fluid and joint regions. This results in the development of chronic inflammatory arthritis, typically in the first metatarsophalangeal, talar, subtalar, ankle, and knee joints. Although less common, an underrecognized feature of gout is extraarticular deposition, such as in the abdomen. This case report describes the presence of a gouty abdominal mass in a 66-year-old male with *no* prior history of gout. We exhibit our imaging findings and discuss the importance of keeping gout in the differential when an unknown abdominal mass is found.

## Introduction

Gout is the most common cause of inflammatory arthropathy, with a global prevalence of 53 million people and a growth rate incidence of 63.44% from 1990 to 2019. It is typically seen as a disease that affects males more than females; however, the number of females affected has grown from 5.3 million to 13.2 million [1]. It has a multifactorial etiology, with genetics, medical comorbidities, and diet all playing a role. Gout occurs via the last step of purine metabolism, where hypoxanthine is converted to xanthine and ultimately creates uric acid. Biochemically, gout manifests due to the oversaturation of urate, with plasma serum levels of 6.8 mg/dL being the upper limit of solubility. When the solubility level is exceeded, the urate turns into urate crystals in the form of tophaceous deposits [2]. Polarized light microscopy is the gold standard for diagnosis, and it will show needle-shaped monosodium urate (MSU) crystals with negative birefringence.

Typically, gout manifests as acute pain, redness, and swelling of the first metatarsophalangeal joint. However, it also occurs in other joints such as the talar, subtalar, ankle and knee [3]. One manifestation of gout that is often overlooked is urate crystal deposition in extra-articular sites. This includes cardiovascular, renal, spinal, ocular, and gastrointestinal. In previous cases, the diagnosis of gout was already established in the patient before the discovery of the extra-articular depositions [4]. In this case report, we discuss a patient with no prior history of gout diagnosis who presented with an abdominal mass that was found to have MSU crystal deposits indicative of primary gout.

## Case presentation

We present a case of a 66-year-old male with a past medical history notable for severe refeeding syndrome, celiac disease, and significant tobacco use with emphysematous presentation on chest CT who presented to the ER in April 2023 with generalized weakness, abdominal pain, bilateral lower leg cramping, and watery, non-bloody diarrhea over the past two weeks. He also endorsed a 20-pound weight loss during the same time period and denied any fever, chills, night sweats, or previous presentations of gout. He denied previous complaints of diarrhea or abdominal pain despite not being adherent to a gluten-free diet due to socioeconomic barriers. Initial ER labs were notable for multiple electrolyte abnormalities, including hypokalemia of 1.8, hypomagnesemia of 1.5, hypophosphatemia of 1.4, troponin elevation of 61 (which later down trended to 55), and alkaline phosphatase elevation to 293. Uric acid levels were found to be surprisingly low, with a value of 2.5. However, in previous visits two and five years ago, his uric acid levels were 7.0 and 7.8 respectively.

**Figure 1:**
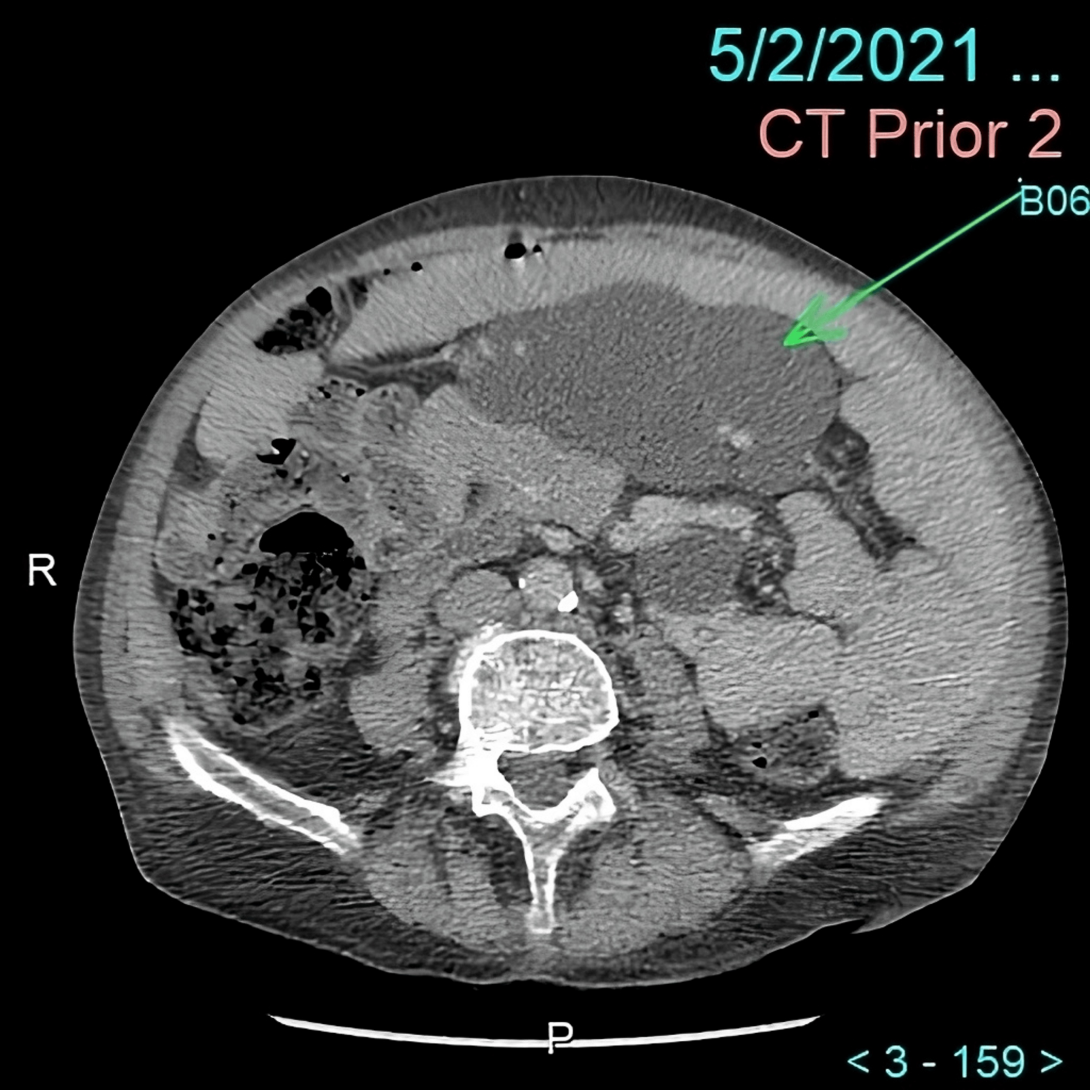
CT abdomen and pelvis acquired after administration of 96 cc IV contrast in May 2021 shows a mesenteric lobulated low attenuating space occupying lesion measuring approximately 12 cm x 6.6 cm with a mean CT number of 11 units (green arrow). There is now bowel obstruction

He was diagnosed with celiac disease in May 2021, at which time abdominal CT notably showed a lobulated mass (Figure 1). A PET-CT was recommended to rule out lymphoma; however, the patient was lost to follow-up and no further workup was done for the mass at that time.

**Figure 2:**
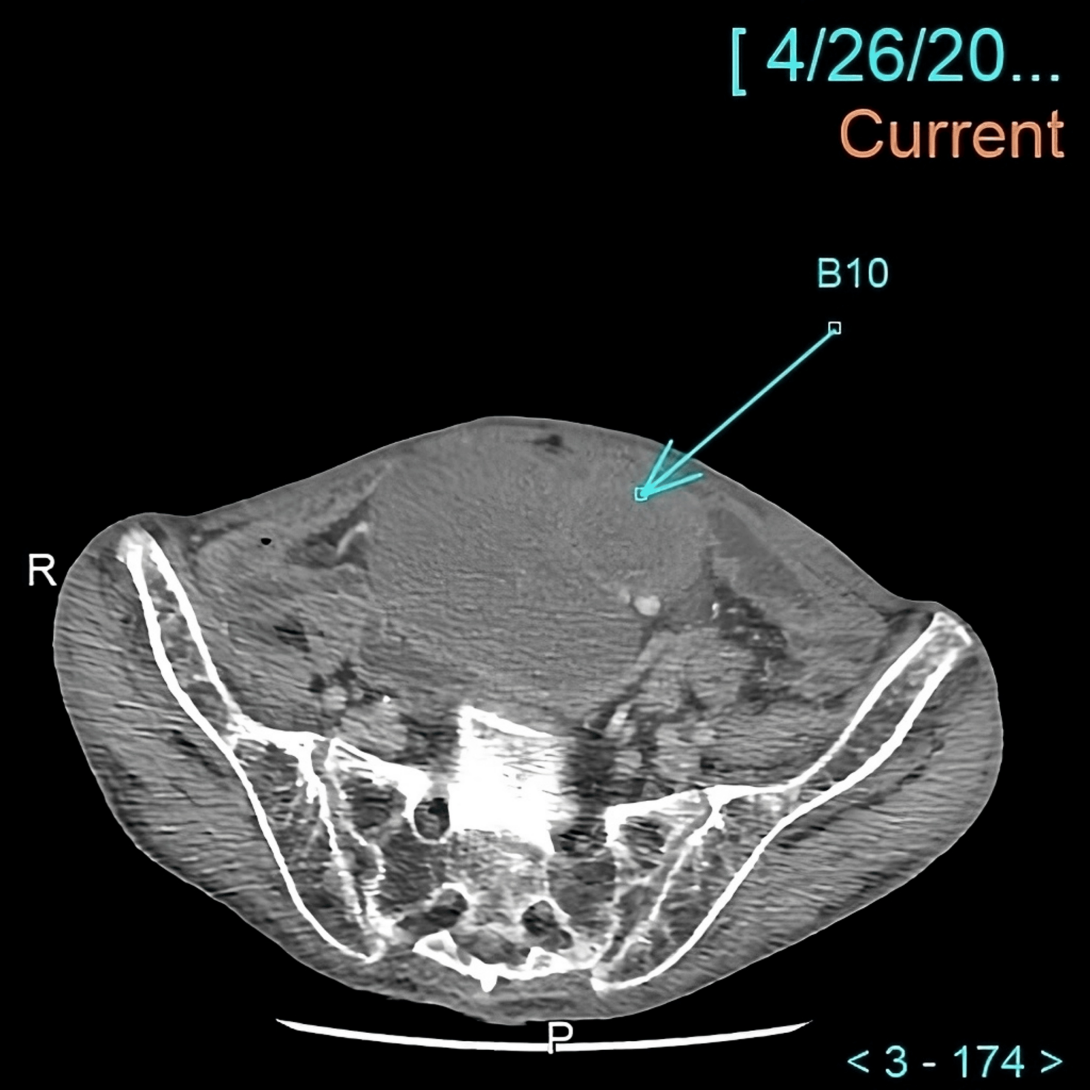
CT abdomen and pelvis done in April 2023 after injection of 80 cc of contrast shows an intraperitoneal lobulated space occupying lesion measuring approximately 11 cm x 7.7 cm with a mean CT number of 37 units (blue arrow).

Abdominal CT was obtained and compared to the CT done in 2021 (Figure 2). Current CT findings were concerning for lymphoma, notably showing dominant cystic lesions from 11 cm x 7.7 cm as well as findings consistent with chronic pancreatitis.

GI was consulted and recommended Creon to treat his malabsorption and endoscopy/colonoscopy given concurrent elevated inflammatory markers in order to rule out enteropathy-associated T-cell lymphoma. The patient denied colonoscopy in 2021 and also refused it during the current hospitalization. Hematology/oncology also expressed concerns for lymphoma, and the patient underwent core needle biopsy of the abdominal mass. Biopsy results revealed numerous needle-shaped crystals with negative birefringence under polarized light, characteristic of the MSU crystal deposits in gout. Rheumatology was then consulted for the concern of extra-articular gout and treatment options were discussed. 

## Discussion

We report a unique case of a primary presentation of gout in an extra-articular setting. There have been cases highlighting the possibility of extra-articular gout deposition, however only a few of them resulted in a first-time diagnosis of gout. Most of the patients in these cases already had a known diagnosis of gout in their past medical history [4,5].

When the patient presented with an abdominal mass and extensive weight loss, the initial concern was enteropathy-associated T cell lymphoma (EATL), which is a rare form of non-Hodgkin lymphoma and has a poor survival prognosis. EATL is also associated with celiac disease, which our patient also had. A previous retrospective study looked at the prognosis of EATL in patients with celiac disease and found 27 of the 37 patients died during follow-up with a median survival of seven months [6]. The severity of EATL makes early diagnosis and treatment highly crucial.

For comparison, we identified other case reports of abdominal manifestation of gout and looked to see what the clinicians suspected when making their differential diagnosis. We found four case reports of tophi within the pancreas that were indistinguishable from malignancy and/or pancreatic pseudocysts on imaging, thus requiring biopsy in order to characterize the mass [4]. Additionally, another case report highlighted a patient who presented with intra-hepatic tophi that warranted biopsy to distinguish from malignancy. The report stressed the importance of histopathological evaluation due to the difficulty of characterizing gouty tophi by imaging. In all of these cases, the patient already had a diagnosis of gout [5].

Interestingly, biopsy might not be the only way to differentiate between gouty tophi and possible malignancy. The use of Dual-Energy CT (DECT) has the capabilities of diagnosing gout in the setting of extra-articular manifestations. This imaging technique uses two energy levels (typically 80 kV and 140 kV) to obtain images and then with the help of mathematical algorithms can help distinguish materials based on their atomic numbers [7]. A previous case series highlights four cases with extra-articular gout deposition and how the use of DECT helped confirm or exclude the diagnosis of gout. The site of deposition included the Achilles tendon in each of the four cases [8]. DECT is beneficial when the site of deposition cannot be aspirated or there is insufficient amount of fluid to aspirate. Additionally, it can aid in diagnosis when clinicians believe there is a high chance of false negative results on aspiration, as in the case of tendons and bursae [9]. A study consisting of 94 patients looked to see the accuracy and sensitivity of DECT in finding uric acid crystals for patients with suspected gout. Two blinded radiologists evaluated DECT images and determined whether the images were positive or negative for uric acid crystals. They used joint aspiration as the reference standard. Both readers had no false negative findings, with a sensitivity of 100% for both readers [10]. Limiting factors to DECT include availability, cost, radiation exposure, and generation of artifacts on the images [9]. The institution that this patient presented to did not have DECT available, thus the next best way of characterizing the mass was through CT-guided biopsy. 

Lastly, it is important to not go off of uric acid levels when thinking about including gout in the differential diagnosis. A retrospective study looked at 30 patients diagnosed with an acute gout attack and found normal uric acid levels in 63.3% of the patients [11]. Additionally, another study with a cohort of 221 gout patients found normal uric acid levels during acute gout attacks were more common in post-surgical episodes, hemodialysis initiation, and in states of higher inflammatory activity. It is thought that the low uric acid levels seen in this patient were secondary to an elevated inflammatory state. Rates of recurrent gout episodes did not differ between individuals with high uric acid levels and normal uric acid levels [12]. Light microscopy showing the monosodium urate crystals is all that is needed to confirm the diagnosis of gout. Thus, low uric acid levels should not sway a clinician’s decision-making regarding the diagnosis of gout. 

## Conclusions

This case report highlights the importance of considering gout in the differential diagnosis when presented with a patient with an abdominal mass. Biopsy of the mass will reveal the monosodium urate crystals that are highly specific for gout. If available, DECT should be done before biopsy to further confirm or reject the diagnosis of gout and lower the risk of biopsy related complications. Further research should look into the possible additional complications extra-articular gout depositions presents to the patient compared to typical gout deposition sites. 

## References

[1] He Q, Mok TN, Sin TH (2023). Global, regional, and national prevalence of gout from 1990 to 2019: age-period-cohort analysis with future burden prediction. JMIR Public Health Surveill.

[2] Dalbeth N, Choi HK, Joosten LA, Khanna PP, Matsuo H, Perez-Ruiz F, Stamp LK (2019). Gout. Nat Rev Dis Primers.

[3] Hainer BL, Matheson E, Wilkes RT (2014). Diagnosis, treatment, and prevention of gout. Am Fam Physician.

[4] Khanna P, Johnson RJ, Marder B, LaMoreaux B, Kumar A (2020). Systemic urate deposition: an unrecognized complication of gout?. J Clin Med.

[5] Ministrini S, Baronio G, Zorzi F, Bercich L, Grazioli L, Molfino S, Portolani N (2019). Unusual presentation of gouty tophus in the liver with subsequent appearance in the same site of HCC: a correlate diagnosis? Case report. World J Surg Oncol.

[6] Malamut G, Chandesris O, Verkarre V (2013). Enteropathy associated T cell lymphoma in celiac disease: a large retrospective study. Dig Liver Dis.

[7] Khanna I, Pietro R, Ali Y (2021). What has dual energy CT taught us about gout?. Curr Rheumatol Rep.

[8] De Vulder N, Chen M, Huysse W, Herregods N, Verstraete K, Jans L (2020). Case series: dual-energy CT in extra-articular manifestations of gout: main teaching point: dual-energy CT is a valuable asset in the detection of extra-articular manifestations of gout. J Belg Soc Radiol.

[9] Chou H, Chin TY, Peh WC (2017). Dual-energy CT in gout - a review of current concepts and applications. J Med Radiat Sci.

[10] Glazebrook KN, Guimarães LS, Murthy NS (2011). Identification of intraarticular and periarticular uric acid crystals with dual-energy CT: initial evaluation. Radiology.

[11] Bădulescu M, Macovei L, Rezuş E (2014). Acute gout attack with normal serum uric acid levels. Rev Med Chir Soc Med Nat Iasi.

[12] Lee JS, Kwon OC, Oh JS, Kim YG, Lee CK, Yoo B, Hong S (2020). Clinical features and recurrent attack in gout patients according to serum urate levels during an acute attack. Korean J Intern Med.

